# Grand challenge of antibiotics resistance: A global, multidisciplinary effort is needed

**DOI:** 10.3389/frabi.2022.984076

**Published:** 2022-08-15

**Authors:** Jianping Xie

**Affiliations:** Institute of Modern Biopharmaceuticals, School of Life Sciences, Southwest University, Chongqing, China

**Keywords:** antibiotics resistance, stakeholder, phage, multidisciplinary, indole, quorum sensing

## Antibiotic miracle is being seriously challenged

The discovery and widespread clinical administration of penicillin (Sykes, 2001) and other antibiotics (Wainwright, 1991) have transformed medicine and civilization. Antibiotics became the cornerstone of modern medicine which greatly prolonged the average life expectancy of civilized nations (Exner et al., 2001; Casanova et al., 2013). However, the misuse and abuse of antibiotics, and the global dissemination of antibiotic-resistance determinants, have rapidly exacerbated the prevalence of antibiotic resistance on a global scale (Zaffiri et al., 2013). Antibiotic resistance or antimicrobial resistance (AMR) is an ever-growing concern for both public health and the global economy. Infections by multidrug-resistant pathogens are increasingly reported across the globe. The drying up of the antibiotics pipeline largely due to the lack of economic incentives and failure of the market mechanism further aggravated this scenario (Spellberg et al., 2015). We will soon face a moment when antibiotics will be futile for some bacterial infections. Resistant infections are predicted to become the leading cause of death which will reach 10 million per year by 2050.

## Multi-prongs are essential to curtailing the upward trend of antibiotic resistance

Great efforts are required to prevent the dire prediction from turning into reality. Tracing the origins of antibiotic resistance, including but not limited to antibiotic-resistant bacteria (ARB), antibiotic resistance genes (ARGs), non-genetic mechanisms of antibiotic response modulation and communication (El-Halfawy and Valvano, 2012), heightened awareness of the risks of overusing antibiotics, improving surveillance, optimizing the duration of antibiotic treatment, a possible shift of prescribing practices, novel antibacterial compounds, and educating more stakeholders are necessary to curb the spread of the ongoing antimicrobial resistance developments that also affect last-resort antibiotics.

Thinking out of the box is essential for all stakeholders, from basic to clinic, from industry to policy making. Appreciation of the multiple roles of antibiotics beyond chemical warfare weapons in natural niches, such as inter-microbial signaling molecules, regulators of gene expression, microbial carbon and nitrogen sources, mediators of host immune response, and a conception paradigm shift of antibiotics, can nourish and promote the design and implementation of integrative antimicrobial stewardship programs (ASPs) (Yap, 2013).

Metabolic byproducts, such as indole (Lee et al., 2010; Wang et al., 2019), indole acetic acid, polyamines, ammonia, cyclic diguanylate (c-di-GMP) (Wang et al., 2011), cAMP (Kwan et al., 2015), 13-methyltetradecanoic acid, and the Pseudomonas quinolone signal, secreted from bacterial cells or present in the bacterial cell milieu, can be infochemicals and modulate bacterial responses toward antibiotics, changing intrinsic resistance to antibiotics and its spread among bacterial cell populations. Further investigation of these metabolic byproducts can inform novel antibiotic targets, especially identification of the key chemical signals mediating increased intrinsic antibiotic resistance.

The role of microbiota shift in antibiotics tolerance, resistance, and persister development is an emerging direction for better control of antibiotics resistance (Liu et al., 2021). More efforts in this aspect are worthwhile.

To tackle antibiotics resistance, One Health-based approach is pivotal to include antibiotics used in both veterinary medicine and human health, livestock growth promoter (Cully, 2014; Silley and Stephan, 2017), transfer of highly mobile ARG across the environment, clinical, and animal-associated bacteria, as well as microbial ecology, such as phage-mediated ARG transfer. This is critical for informing policies aimed at sustainable development. Timely and suitable communication about the problem of resistance is essential to solving antibiotics resistance too. The adverse consequences of antibiotic resistance include health care systems and society, it is also very important to have the stakeholders of both macroeconomic and microeconomic in the panelist to mitigate the societal costs of antibiotic resistance, to avert the antibiotics “tragedy of the commons”.

## Integrative efforts are necessary

Cross-section studies are urgently needed to identify the gaps where future primary research should focus on for a sustainable antibiotics pipeline, such as integrated multi-omics studies to understand the intricate mechanisms of action of current antibiotics, cost-effective methods to effectively monitor the distribution, spatiotemporal dynamics of antibiotic resistance genes, their proliferation, dissemination, and influencing factors in environmental ecosystems to reduce the resistance, assessment, and control of the ecological risks of antibiotic resistance.

New technologies, such as high-throughput sequencing, which can simultaneously sequence thousands of antibiotic-resistant gene targets representing a full-spectrum of antibiotic resistance classes, are most desirable, especially when portable, and can alleviate some of the obstacles hindering the antibiotics resistance survey.

A largely ignored field is the elimination the transmissible resistances in industrial cultures from the starter industries, which can promote the process control and the safety screening of commercial cultures.

Repeated exposure of microbes to chemicals other than antibiotics, such as triclosan (Leyn et al., 2021), a biocide commonly used in household and personal care cleaning agents to prevent microbial growth and an emerging pollutant, can increase the selection pressure for antibiotic resistance and cross-resistance. The triclosan can select and enrich mutations in genes that encode bacterial efflux pumps and fatty acid biosynthesis, which can expel antibiotics and confer broad-spectrum tolerance to antibiotics. To avoid more serious proliferation and dissemination of various resistance genes, provident management during the pandemic era.

Epigenetic factors such as DNA adenine methyltransferase (dam) are involved in replication, mismatch repair, and transposition, and they play a role in safeguarding against antibiotic stress. More in-depth study in this not yet fully defined field can provide further mechanistic insights into antibiotics resistance and accelerate the discovery of new antibiotics targets. Novel mechanism related to antibiotic resistance, such as Lysine 2-hydroxyisobutyrylation (Khib) protein posttranslational modification conserved in eukaryotes and prokaryotes, is rather new to this field (Zheng et al., 2021).

Quorum sensing (QS) is a major regulatory and cell-to-cell communication system for bacterial social adaptation, virulence factor production, biofilm formation, and antibiotic resistance. Many metabolites are engaged in QS. Indole is one of those intensively studied. Indole functions as an intercellular, interspecies, and interkingdom signaling molecule, controlling diverse aspects of bacterial physiology. Indole also regulates various bacterial phenotypes important for antimicrobial resistance. Quorum sensing inhibitors (QSIs) are explored as promising antibiotic substitutes, which can be individually or jointly used with traditional antibiotics (Ning et al., 2021). The discovery of new antibiotic adjuvants is an attractive option for overcoming antibiotic resistance.

Biofilm formation of pathogens is one of the major global challenges to control nosocomial infections due to their high antimicrobial resistance. Chemicals that control biofilm-associated infections can be an efficient strategy to overcome this resistance. Biofilm is also tangled with quorum sensing. An integrative probe into their intricate interaction might unveil more novel targets for antibiotics resistance control.

The population dynamics during the development of antibiotic-resistant strains are extremely poorly addressed. Identification of molecules underlying bacterial general stress responses and antibiotic resistance can establish new measures against a population-based resistance mechanism, which seems to be more beneficial to the control of antibiotic resistance.

Microbial communities are shaped by bacteriophages through predation and lysogeny (Cairns et al., 2017; Lin et al., 2017). The role of phage, and microbial community in antibiotic resistance is very new to this field. A better understanding of the interactions between the processes across different types of environments is key to elucidating how phages mediate microbial competition and to designing efficient phage therapies in combination with antibiotics to effectively mitigate the damage of the resistant bacteria (Diacon et al., 2022). Reversing bacterial resistance to antibiotics by phage-mediated delivery of dominant sensitive genes is actively explored (Edgar et al., 2012; Sousa and Rocha, 2019). Many are still unknown about the interactions between phage, bacteria, and the human host. The time to revitalize phage therapy seems to be rapidly approaching, including using bioengineered phages or purified phage lytic proteins as either an alternative or a supplement to antibiotic treatments (Projan, 2004). Phage-antibiotic combination (PAC) therapy (Abedon, 2019; Luo et al., 2022), a promising alternative to control pathogenic bacteria infections, particularly antibiotic-resistant bacteria is attractive to recalcitrant infections. Phage display platforms enabling rapid identification of peptide probes for specific bacterial strains, with an aim for phage-inspired antibiotics are harnessed by many start-up biomedicine companies (McCarthy et al., 2018).

Developing persister-targeting antibiotics is a much-needed direction (Vega et al., 2012), which can interfere with cellular components, such as tryptophanase, which regulates pH homeostasis (Goode et al., 2021).

Stewardship of antibiotics from ONE HEALTH big picture is indispensable to address the crisis of antibiotics resistance and for a healthier world; all stakeholders in this aspect have an aim to sustain the development of antibiotics. Interdisciplinary efforts related to antibiotics resistance, medical, biology, public policy, genetic basis, clinical trial, biochemical, chemical, microbiological, and pharmacological studies, novel physical, chemical, biochemical, microbiological, or pharmacological methods for antibiotic resistance detection, clinical, epidemiological, or molecular characterization of antibiotic resistance, Deep Learning Approach combined with experimental validation, and meta-analysis are crucial.

Though the threat of a pandemic of antibiotic-resistant infectious diseases is ever-growing, with the input from all stakeholders, we are confident that we can find new and better antibiotics and ways to defeat antibiotic-resistant pathogenic bacteria.

## Author contributions

The author confirms being the sole contributor of this work and has approved it for publication.

## Funding

The work was supported by funding from NSFC 82072246 and 82211530059.

## Conflict of interest

The author declares that the research was conducted in the absence of any commercial or financial relationships that could be construed as a potential conflict of interest.

## Publisher's note

All claims expressed in this article are solely those of the authors and do not necessarily represent those of their affiliated organizations, or those of the publisher, the editors and the reviewers. Any product that may be evaluated in this article, or claim that may be made by its manufacturer, is not guaranteed or endorsed by the publisher.
